# Effects of Visual Predictive Information and Sequential Context on Neural Processing of Musical Syntax

**DOI:** 10.3389/fpsyg.2018.02528

**Published:** 2018-12-13

**Authors:** Hana Shin, Takako Fujioka

**Affiliations:** ^1^Department of Music, Center for Computer Research in Music and Acoustics, Stanford University, Stanford, CA, United States; ^2^Stanford Neurosciences Institute, Stanford University, Stanford, CA, United States

**Keywords:** electroencephalography, music syntax processing, auditory cortex, predictive coding, ERAN, tonality

## Abstract

The early right anterior negativity (ERAN) in event-related potentials (ERPs) is typically elicited by syntactically unexpected events in Western tonal music. We examined how visual predictive information influences syntactic processing, how musical or non-musical cues have different effects, and how they interact with sequential effects between trials, which could modulate with the strength of the sense of established tonality. The EEG was recorded from musicians who listened to chord sequences paired with one of four types of visual stimuli; two provided predictive information about the syntactic validity of the last chord through either musical notation of the whole sequence, or the word “regular” or “irregular,” while the other two, empty musical staves or a blank screen, provided no information. Half of the sequences ended with the syntactically invalid Neapolitan sixth chord, while the other half ended with the Tonic chord. Clear ERAN was observed in frontocentral electrodes in all conditions. A principal component analysis (PCA) was performed on the grand average response in the audio-only condition, to separate spatio-temporal dynamics of different scalp areas as principal components (PCs) and use them to extract auditory-related neural activities in the other visual-cue conditions. The first principal component (PC1) showed a symmetrical frontocentral topography, while the second (PC2) showed a right-lateralized frontal concentration. A source analysis confirmed the relative contribution of temporal sources to the former and a right frontal source to the latter. Cue predictability affected only the ERAN projected onto PC1, especially when the previous trial ended with the Tonic chord. The ERAN in PC2 was reduced in the trials following Neapolitan endings in general. However, the extent of this reduction differed between cue-styles, whereby it was nearly absent when musical notation was used, regardless of whether the staves were filled with notes or empty. The results suggest that the right frontal areas carry out the primary role in musical syntactic analysis and integration of the ongoing context, which produce schematic expectations that, together with the veridical expectation incorporated by the temporal areas, inform musical syntactic processing in musicians.

## Introduction

A primary feature in much of Western music is the concept of *tonality*, or “the orientation of melodies and harmonies toward a referential (or tonic) pitch class” (Hyer, 2001). The regularities governing the arrangement of tonal functions among different harmonies are considered to comprise a part of “musical syntax,” and the implicit understanding of musical syntax is thought to be acquired at an early age and throughout development, through cultural exposure to music (Trainor and Trehub, 1994; Koelsch et al., 2003). Knowledge about musical *schemata*, defined as rules that govern the organization of sounds in music, remains implicit for non-musicians, while it becomes explicit for musicians with education and training. Nevertheless, musicians and non-musicians alike can use this *schematic* expectation and detect violations when listening to music (Koelsch et al., 2000; Tillmann, 2005). When listeners process upcoming events, the processing time is faster if the events match the *schematic* expectation (Bharucha and Stoeckig, 1986; Bigand and Pineau, 1997; Tillmann et al., 2003b). However, if the preceding context is atonal or in violation of tonal rules, distraction or interference occurs (Tillmann et al., 2008). This suggests that online syntactic processing dynamically updates the scheme by incorporating newly heard events. Listeners also use *veridical* expectations for processing upcoming musical events, based on specific knowledge about the nature and context of those events. For example, listeners could anticipate an abrupt key change in a song when they are familiar with the song structure, even if such change might not fit with the schematic expectation related to the musical events leading up to it. These two types of expectation interact with each other in determining the overall expectation processing in music, for both listeners and performers. During sight-reading of an unfamiliar piece, musicians could anticipate an upcoming harmonic event based on schematic expectation, while they may overlook an unusual event that does not match the schematic expectation and instead requires veridical expectation based on familiarity (Sloboda, 1984).

To date, numerous studies have examined neural processing of musical syntax with event-related potentials (ERPs), investigating how the brain processes events that violate schematic expectations. Koelsch et al. (2000) designed a paradigm with syntax-violating chords occurring amidst tonal chord progressions, revealing an early right anterior negativity (ERAN) around 150–250 ms after their onset. The ERAN can be elicited preattentively (Koelsch et al., 2002c, 2007) in both musicians and non-musicians, with larger amplitudes in musicians (Koelsch et al., 2000, 2002b); is sensitive to the degree of musical expectancy built up by the tonal context (Koelsch et al., 2000, 2004; Leino et al., 2007); and is present in children (Jentschke et al., 2005, 2008; Jentschke and Koelsch, 2009; Trainor, 2012). Brain imaging, magnetoencephalography (MEG), and brain lesion studies have pointed to Brodmann area (BA) 44 of the inferior frontal gyrus (IFG) as the primary generator of the ERAN response, with additional contributions from the auditory cortical area, such as the superior temporal gyrus (STG), the superior temporal sulcus (STS), and the middle temporal gyrus (MTG), as well as the ventrolateral premotor cortex (Maess et al., 2001; Koelsch et al., 2002a; 2005; Tillmann et al., 2006; Garza Villarreal et al., 2011; Sammler et al., 2011). Despite what its name suggests (the early *right* anterior negativity), the ERAN is often observed bilaterally (Koelsch et al., 2000; Maess et al., 2001; Garza Villarreal et al., 2011; Brattico et al., 2013), with considerable overlaps with language-specific ERPs such as the early left anterior negativity (ELAN) (Sammler et al., 2013), and with somewhat unclear patterns of laterality depending on factors such as gender (Koelsch et al., 2003) and expertise (Kalda and Minati, 2012; Fitzroy and Sanders, 2013). Overall, the ERAN response seems to involve a complex bilateral frontotemporal network that serves syntactic processing in both language and music in a partially domain-general manner.

An interesting question arises as to whether the ERAN is involved in the processing of violations of schematic expectations based on musical syntax representation (Bharucha, 1992), or violations of expectations in general about the predictability of events, regardless of the type of information from which the prediction is drawn, or whether such information is conveyed implicitly or explicitly. In Experiment 4 of Koelsch et al. (2000), the ERAN was still elicited when trials ending with the Neapolitan sixth chord were presented with a 50% probability, but with a smaller amplitude compared to when they were presented with a 25% probability. However, the predictive information in this setup was derived implicitly from stimulus probability, and it may be processed differently when used explicitly and consciously by the listener. Along these lines, behavioral studies have shown that the veridical expectation about the critical chord does not abolish the priming effect for schematically expected harmony, even when such expectation is formed through a preview of the critical chord beforehand (Justus and Bharucha, 2001) or familiarization with a less-expected musical structure (Tillmann and Bigand, 2010). Furthermore, recently, Guo and Koelsch (2016) found that providing predictive visual cues (colored fixation crosses on the screen) elicited the ERAN earlier in both musicians and non-musicians, but did not influence the amplitude. Similarly, a study of audiovisual melodic processing in musicians found that an ERAN-like peak was larger in amplitude when melodies had harmonically “unstable” endings than when they had “stable” endings, despite the availability of the visual score that would have allowed the listeners to build veridical expectations about the melodic endings (Schön and Besson, 2005). Thus, it appears that ERAN responses represent auditory processing based on schematic expectations, which may be resilient to veridical expectations shaped by implicit knowledge about event probabilities or explicit knowledge about upcoming events given by visual cue information.

It is still underexplored how schematic processing is influenced differently by the type of information that is used to form the veridical expectation. For musical sound processing, when the expectation formed by the visual score on the upcoming auditory stimulus was violated (i.e., audio-visual mismatch), early stages of auditory processing, indexed by ERPs such as N1 and P3, were modulated (Tervaniemi et al., 2003; Schön and Besson, 2005; Yumoto et al., 2005). Although this finding was generally replicated in non-musicians viewing visual symbols and animation easily associable to auditory stimuli (Widmann et al., 2004; Aoyama et al., 2006; Laine et al., 2007; Horváth et al., 2011), it has been shown that a visually presented musical score can trigger auditory imagery in experienced musicians (Banton, 1995; Brodsky et al., 2003). Musical notation provides unambiguous information about upcoming auditory events for musicians who can read scores, compared to words or symbols that need context-specific associations to specify the events. Moreover, schematic tonality processing plays an important role in music reading, as found in sight-reading studies with musicians, in which pitch errors were more frequently made in an atonal context than in a tonal one (Fine et al., 2006), and printing errors were overlooked when the resulting notation matched expected harmonic features (Sloboda, 1976; Wolf, 1976). Thus, it is possible that veridical expectation based on musical scores may influence tonal syntax processing through auditory imagery in music score reading, a potentially different route compared to those involving visual information unrelated to music. To our knowledge, there is no study comparing how these two types of visual information might influence tonality processing in the brain differently. In an interesting visual perception study, audio-visual mismatch between a heard melody and a seen melody on a score affected resolution of visual binocular rivalry only in those who could read musical scores, but not when they were supposed to ignore the score (Lee et al., 2015); this suggests that extra attentional processes based on explicit predictive information are called upon for auditory-visual processing.

Another factor could influence the interaction between schematic and veridical expectation in tonal processing: evaluation of the consequence of the schematic expectation. In other words, schematic expectation may well depend on how strongly the sense of tonality is internally held on a moment-to-moment basis *after* the fact, whether or not the expectation has been met. As listeners go through unfolding musical events, the musical schemata are continuously used to analyze an incoming event and synthesize the cumulative representation of the current music. This representation is dynamically updated by incorporating the current event, and is used to form an expectation toward the next event, along with the long-term knowledge of musical schemata. In fact, many other studies outside of music investigating processing of incongruity between a target and its priming context found sequential effects, where the nature of the *previous* trial affects the degree of incongruity processing of the *current* trial. What is the influence of having already elicited an ERAN response, upon the upcoming syntax processing? In processing stimulus- and response-related conflict, as in the Stroop and Simon tasks, the reaction time is typically slower in not only the incongruent trials but also the congruent trials that follow incongruent trials, even if repetition priming is removed. This behavioral sequential effect was reflected in the ERP components N2 and P3 and slow potentials, considered to be involved in “conflict adaptation” processes (Ullsperger et al., 2005; Larson et al., 2011, 2014). Sequential effects have also been observed in the MMN/N2-like negativity in oddball paradigms, when standard trials immediately followed a deviant trial (Sams et al., 1983, 1984). Interestingly again, in oddball paradigms, stimulus-specific visual cue information reduced the P3a, indicating decreased involuntary attention allocation (Horváth et al., 2011). If, similarly, the ERAN response reflects modulation of online processing of an incongruity between the preceding tonal context and the critical chord, the ERAN may be sensitive to the previous trial type, an effect that may in turn interact with the veridical expectation formed by the visual predictive information. We could not find any previous studies that examined this involving the ERAN.

As mentioned above, harmonic syntax processing involves the inferior frontolateral cortex in the IFG around BA 44 as the main contributor, with additional contributions from multiple regions in the bilateral auditory cortical area such as the STG, the STS, and the MTG, as well as the medial frontal cortex and the thalamus (Maess et al., 2001; Koelsch et al., 2002a, 2005; Tillmann et al., 2006; Garza Villarreal et al., 2011; Sammler et al., 2011). The primary role that the IFG plays in ERAN generation is supported by evidence showing that the ERAN is abolished in patients with a lesion in the IFG (Sammler et al., 2011), and in participants under deep sedation that effectively shuts off frontal lobe functions (Koelsch et al., 2006). Together with findings on linguistic processing (Friederici, 2002), the current data suggest that this area is firmly responsible for analyzing and predicting sequential auditory information. On the other hand, the temporal lobe’s contribution in harmonic processing has also been consistently observed (Koelsch et al., 2005; Tillmann et al., 2006). Tillmann et al. (2006) noted that, “our data pattern further suggests that musical syntax processing includes not only frontal opercular and anterior insula activation, but the integration of these frontal areas in an overall neural system.” The distinction of functional roles between the temporal and frontal areas has been associated with the difference between the physical- and abstract-feature MMN, which is involved in the processing of online contexts, and the ERAN, which is based on long-term knowledge (Koelsch, 2009). Regarding schematic and veridical expectations, explicit expectation reduces the amplitude of the MMN originating in the temporal lobe (Todorovic and de Lange, 2012; Chennu et al., 2013). Thus, if veridical information interacts with schematic operation in harmonic syntax processing, these two areas may operate differently in ERAN generation. Specifically, the ERAN in the frontal area might be less influenced by the veridical expectation than the one in the temporal areas.

The goal of the present study was threefold. The first aim was to test the effect of different types of visual predictive information regarding the syntactic validity of the last chord in a sequence, on schematic processing as reflected by the ERAN. The audio stimuli were presented in conjunction with one of four types of visual stimuli: two types of informative cues in the form of a word (either “regular” or “irregular”) or a musical score of the whole chord sequence; and two types of non-informative visual controls in the form of empty musical staves or a blank screen. The second goal of the study was to examine whether schematic processing is dynamically affected by the consequence of the expectation evaluation and how this effect interacts with the veridical expectation, by looking into the sequential effect between trials. Here, we presented Tonic-ending and Neapolitan-ending trials in a 50/50 ratio, but with all the trials in the same key for an entire block. This was done in order to investigate whether or not the ERAN responses would differ when following a Tonic-ending trial compared to when following a Neapolitan-ending trial, and how this difference would further influence the processing of visual predictive information. The final aim of the study was to examine whether the frontal and temporal ERAN generators described in previous work are related differently to processing of these three factors: cue predictability, visual cue style, and sequential context. We hypothesized that, if schematic and veridical expectation influences the ERAN, it will vary according to the presence or absence of visual predictive information, with possible differences in latency and/or amplitude between the different cue styles. Furthermore, if the schematic expectation strength varies dynamically, the influence of veridical expectation on the ERAN might also vary according to the type of the previous trial. Finally, we expected that the ERAN could be separated into frontal and temporal components, with distinct spatio-temporal dynamics. These components may differ in terms of sensitivity to veridical expectation and visual cue types. Thus, we examined the PCs of the entire auditory evoked response and analyzed the time course of neural activities projected onto the different components with respect to cue predictability, cue styles, and preceding context regarding tonal congruence.

## Materials and Methods

### Subjects

Nineteen musically trained subjects participated in this study. After one subject was excluded because of excessive noise in the data, the final sample consisted of 18 musicians (8 females) between 18 and 35 years of age (*M* = 25.6, *SD* = 3.77). All subjects had practiced one or more instrument(s) regularly for more than 9 years with formal training (*M* = 13.7, *SD* = 4.44), and were proficient in reading Western musical notation, including grand staves (i.e., a pair of staves in which the top part follows the treble clef and the bottom follows the bass clef, typically used for keyboard playing). All but one subject were right-handed, and none of the subjects reported having had hearing or vision problems. The subjects gave informed consent to participate after having been completely informed about the nature of the study. All experimental procedures were approved by the Stanford Institutional Review Board.

### Stimuli and Task

The stimuli were five-chord sequences, presented in four-part harmony, partially adopted from the initial ERAN paradigm of Koelsch et al. (2000). The first chord was always the Tonic in root position. The second and third chords were in three different combinations following standard Western harmonic syntax: the first-inversion Tonic followed by the subdominant, the subdominant followed by the second-inversion Tonic, or the submediant followed by the dominant. The fourth chord was always the dominant seventh in root position, and the last chord, or the *critical chord*, was either the Tonic (regular) or the Neapolitan sixth (irregular) chord, occurring at a 50/50 ratio. The three different sequence types (as determined by the second and third chords) were presented with different voice-leading to avoid potential neural responses to specific expectations regarding melodic continuation, and each was repeated 23 times in a block, resulting in a total of 69 sequences ending with the Tonic chord and 69 sequences ending with the Neapolitan chord. The stimuli were created from digitally recorded piano samples at a sampling rate of 44,100 Hz with 16-bit depth.

**FIGURE 1:**
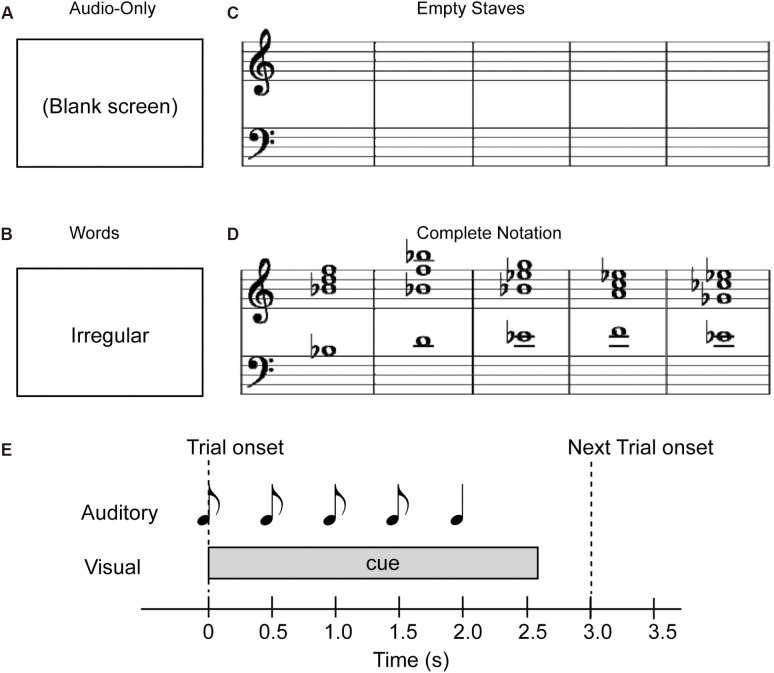
Examples of visual cue stimuli used for the study. **(A)** Audio-only, **(B)** Word, **(C)** Empty Staves, and **(D)** Complete Notation conditions. Note that in terms of cue predictability, the Audio-only and Empty Staves (top) conditions are referred to as “non-informative” cues, while the Word and Complete Notation (bottom) conditions are considered “informative” cues. Also, in terms of the cue style, the Audio-only and Word conditions (left) are considered “non-musical” and the Empty Staves and Complete Notation conditions (right) are considered “musical.” **(E)** Trial structure with auditory and visual stimulus.

A block of 145 sequences in total was conducted twice, in two different keys (A major and B-flat major), for each of the four visual cue conditions as described below, for each subject. This block included 7 timbre-deviant targets to maintain the participants’ vigilance. These targets were always derived from Tonic-ending sequences, created similarly, from digitally recorded samples of the marimba, celesta, and harpsichord. The corresponding visual stimulus for the timbre-deviant targets was the same as that of the other trials in a given block. The first four chords in the sequence each lasted for 500 ms, while the fifth chord lasted for 750 ms and was followed by an additional 250 ms of silence before the onset of the next sequence, thereby making each trial 3 s long as shown in Figure 1E.

Four different types of visual stimuli were examined in the present study: (i) a blank white screen for the “Audio-only” condition, (ii) a word representing the quality of the last chord (“regular” for the Tonic, and “irregular” for the Neapolitan) for the “Word” condition, (iii) empty musical staves with five measures for the “Empty staves” condition, and (iv) the full musical notation of the sequence for the “Complete notation” condition. These four visual stimuli are depicted in Figures 1A–D. The notation included no time or key signatures, such that all five chords were depicted in whole notes occupying each of the five measures, and accidentals were marked for each chord. Note that, since we used either the key of A major or B-flat major in a given block, accidentals appeared in most of the chords including the last one, regardless of whether they were in Tonic or Neapolitan trials. All visual stimuli were placed at the horizontal and vertical center of the screen. As shown in Figure 1E, the audio and visual stimuli were organized on the timeline such that a visual stimulus was presented at the onset of the first chord of each trial, and stayed on the screen until 750 ms after the onset of the fifth chord. There was another condition presenting partially completed notation, which was originally designed for a different purpose; this condition was not analyzed in the present study. This block was also repeated twice, thus making a total of 10 blocks. The order of experimental blocks was counterbalanced between participants. The total measuring time for the 10 blocks was 72.5 min.

### EEG Recordings

The EEG apparatus consisted of a whole-head, 64-channel Neuroscan Quik-Cap (10–20 system), a SymAmpRT amplifier, and the Curry 7 acquisition software (Compumedics Neuroscan Inc., El Paso, TX, United States). On the Quik-Cap, a reference electrode was placed at a central midline between Cz and Cpz. Vertical and horizontal electrooculograms (EOG) were also recorded through additional bipolar electrode pairs. During the recording, the data were low-pass filtered online at 200 Hz and sampled at 500 Hz. A trigger-signal corresponding to the onset of the first chord of each sequence was also recorded as part of the EEG data.

The recordings were performed in a seated position in a sound-attenuated and electrically shielded room. The audio stimuli were delivered through insert stereo earphones (ER-1, Etymotic Research, Elk Grove Village, IL, United States), and the visual stimuli were presented straight ahead of the participants at eye level on a monitor located at a distance of approximately 1.2 m. Participants were asked to attend to both audio and visual stimuli, with their eyes either fixated on the center of the screen, or moving along the musical notation, if any, with the unfolding chord sequence. Participants were informed of the presence and meaning of the “regular” and “irregular” chords, and were given the task of detecting the timbre-variant targets with button presses. Participants’ compliance was verified via visual monitoring through a glass window from the adjacent control room.

### Behavioral Data Analysis

Participants’ response to the timbre targets was registered and stored with the EEG continuous data, and extracted offline. The hit-rate was calculated as the ratio of the response against the number of targets for each condition, while mean reaction time across valid responses was calculated in each condition after excluding the trials where the reaction time differed by more than 2 standard deviations from the overall mean. This resulted in no more than 1 trial rejected in each block per participant where the target occurred 7 times, for a total of 4.09% of the overall targets across participants. Note that this task was required to keep the participants’ attention on the sound and assess their overall alertness between conditions. The performance was not related to harmonic syntactic processing, as all the targets were Tonic-ending sequences. The hit-rate and mean reaction time were examined through a two-way repeated measures ANOVA using two within-subject factors of Cue-predictability (informative vs. non-informative) and Cue-style (musical vs. non-musical).

### EEG Data Analysis

After the recording session, the data in the EEG channels were re-referenced to the common average in the Curry software. The EEG data was preprocessed with routines from the Brainstorm toolbox (Tadel et al., 2011). For each participant, eye artifact events based on vertical and horizontal EOG were detected and modeled via signal-space projection in Brainstorm and removed from the continuous data. Thereafter, the data were band-pass filtered between 1 and 40 Hz, and epochs from -200 ms to 3,500 ms with respect to the onset of the first chord of each sequence were extracted. Additionally, channels with peak-to-peak voltage differences exceeding ± 70 μV were rejected from individual epochs. As this rejection was performed on an individual-channel basis, the number of trials has been kept the same as the original number in the blocks.

The epochs were then averaged separately for Tonic-ending and Neapolitan-ending cases for each of the four visual cue conditions and the two preceding context conditions. To assess the ERAN responses, the baseline was re-adjusted using a 50 ms interval after the onset of the fifth chord (2,000 ms after the onset of the first chord of the sequence). This re-adjustment was performed to minimize the influence of the ongoing auditory evoked responses from the fourth chord, by reducing their amplitude almost to zero at the onset of the fifth chord. Trials were averaged separately based on the preceding trial type (Tonic-following or Neapolitan-following) and the current trial type (Tonic-ending, or Neapolitan-ending) for each of the four visual conditions. Thereafter, to examine the ERAN response, difference waveforms were obtained by subtracting the Tonic-ending ERP from the Neapolitan-ending ERP for each preceding context condition.

First, the ERP waveforms and difference waveforms in the frontocentral scalp areas were obtained by averaging 9–10 electrodes from three areas: frontocentral left (*fcl*: AF3, F7, F5, F3, FT7, FC5, FC3, T7, C5, C3), frontocentral midline (*fcm*: F1, Fz, F2, FC1, FCz, FC2, C1, Cz, C2), and frontocentral right (*fcr*: AF4, F4, F6, F8, FC4, FC6, FT8, C4, C6, T8). The amplitudes of the ERAN and P3a peaks in each visual condition and each preceding context condition were extracted as a mean value within a time window defined by the two time points around the grand average peak latency of the negativity for the ERAN and the positivity for the P3a, where half of the peak-to-peak amplitude to either side of the neighboring peak was reached. This yielded time windows of 88–178 ms for the ERAN and 178–340 ms for the P3a. This approach, which is based on the grand average waveform across conditions, follows the recommendation by Keil et al. (2013) for finding a region of interest without selection bias to examine the difference between conditions. The ERAN and P3a amplitudes across different conditions were statistically examined by a repeated measures analysis of variance (ANOVA) with four within-subject factors of Cue-predictability (informative vs. non-informative), Cue-style (musical vs. non-musical), Previous-trial (Tonic vs. Neapolitan), and Scalp-area (Left, Midline, Right). For conditions in which Mauchly’s Test of Sphericity was significant, the Greenhouse-Geisser correction of degrees of freedom was applied for the corresponding *F*-test. *Post hoc* tests were conducted by paired *t*-tests, with the Bonferroni correction.

Further, a principal component analysis (PCA) using singular value decomposition (SVD) (Lagerlund et al., 1997) was performed on the grand averaged auditory evoked response of all 64 channels from the Audio-only condition, in order to extract components related to only auditory processing. The entire epoch, including the responses to the first through fifth chords, was averaged across Tonic- and Neapolitan-ending trials and across all participants. This response was arranged as a two-dimensional matrix of channels by samples, and SVD was applied to yield a set of paired spatial and temporal components, i.e., for each component a topographic map and its corresponding time course. The resulting 1st and 2nd principal components (PCs) accounted for over 90% of the variance. These two PCs were used as spatial filters to extract the time course of component activities. Specifically, difference waveforms in all different conditions were projected onto these two PCs in order to investigate neural activities related to auditory processing separately from those related to visual processing. Such a spatial-filtering approach has been applied successfully in earlier works using PCA (Schaefer et al., 2013; Tremblay et al., 2014) and those using other decomposition methods such as independent component analysis (Makeig et al., 1997; Jung et al., 2001) to investigate time courses of spatially-filtered activities in the MEG/EEG channel space or source space (Fujioka et al., 2012, 2015). Again, the amplitudes of the ERAN and P3a peaks were computed as a mean value in the time window around the grand average peak latency defined by half of the peak-to-peak amplitude to the neighboring peak, in this case, for each PC1 and PC2. This yielded time windows of 88–182 ms for PC1 and 120–230 ms for PC2 of the ERAN, and 182–346 ms for PC1 and 230–394 ms for PC2 of the P3a.

Peak latency of the individual ERAN and P3a responses in the fronto-central midline electrode group for all previous-trial types and all visual conditions was sought as the most negative and positive point of the difference waveform within a time window of 50–250 ms and 120–550 ms, respectively. When the same search was applied to PC1- and PC2-based waveforms, some cases turned out ambiguous. To avoid misidentifying one of the spurious local minima or maxima as a “peak,” the search was re-conducted with these waveforms after a low-pass filter at 20 Hz was applied. Note that the edge effects from filtering did not influence the data around the fifth chord onset, because of the original epoch window spanning -0.2–3.5 s from the 1st chord of the sequence where the fifth chord occurred at 2.0 s. This resulted in a small number of individuals who did not show the local minima (for ERAN) or maxima (for P3a). This was likely related to the lack of responses in some conditions as shown in the amplitude analysis described above. Therefore, these were treated as missing values and replaced with the average value from the other participants. The number of the missing values was 30 out of 288 ( = 18 subjects × 2 trial types × 4 conditions × 2 PCs) cells for ERAN, and 5 out of 288 for P3a. The largest number of missing values within one condition was 5 out of 18 subjects for ERAN in PC2 and Neapolitan-following, Audio-only condition.

These PC-based ERAN and P3a amplitudes, as well as the peak latency measures, were statistically examined with a repeated measures ANOVA with three within-subject factors of Cue-predictability (informative vs. non-informative), Cue-style (notation vs. non-notation), and Previous-trial (Tonic vs. Neapolitan), separately for each PC. The Greenhouse-Geisser correction was used when Mauchly’s Test of Sphericity was significant. *Post hoc* tests were conducted by paired *t*-tests at a significance level of α = 0.05, as all factors had only two levels.

Finally, a source analysis was performed to examine the relative contribution of bilateral temporal lobe sources and a right frontal source, using Brain Electric Source Analysis (BESA) (BESA Research, version 6.1, BESA GmbH, Grafelfing, Germany) (Berg and Scherg, 1994). We took the seeding location approach, which has been widely used for ERPs to observe temporal dynamics of the source activities in the regions of interest (Heinze et al., 1994; Opitz et al., 1999; Crottaz-Herbette and Menon, 2006). Specifically, three locations were taken from the findings described in an earlier fMRI study (Koelsch et al., 2002a) by averaging all the locations in the left temporal lobe, all the locations in the right temporal lobe, and all the locations in the right frontal area. This resulted in three Talairach coordinates in medial-lateral (from left to right), posterior-anterior (from back to front), and inferior-superior (from bottom to top) directions: Left-auditory cortex (AC): -48.2, -26, 15; Right-AC: 49, -30.6, 15; Right-BA44/6: 44.5, 13.5, 18. These locations were added in BESA as regional sources and applied to the grand average auditory evoked responses from which the two PCs were derived, then converted to three single dipoles (e.g., orientation fit). This yielded a Goodness-of-Fit (GOF) of 88.56%, in which the best value was 95.18% around the P2 peak after the 1st chord onset. These regional sources were also applied to the PC1 and PC2 topographic maps and converted to three single dipoles. The strength of each source was extracted from the fit results.

## Results

### Behavioral Performance

Table 1 indicates the hit-rate and mean reaction time of the participants’ responses to the timbre-targets. A two-way repeated measures ANOVA using two within-subject factors of Cue-predictability (informative vs. non-informative) and Cue-style (musical vs. non-musical) revealed no main effects or interaction, confirming that the alertness of the participants was stable across different visual-cue conditions, thus ensuring the integrity and compatibility of the EEG data.

**Table 1:** Behavioral performance for the timbre-target detection task.

Cue-style	Non-musical		Musical	
		
Cue-predictability	Non-informative (audio-only)	Informative (word)	Non-informative (empty-staves)	Informative (complete-notation)
Hit rate (%)	87.15	90.54	88.70	90.13
S.E.M	3.41	2.58	3.16	3.17
Reaction time (s)	1.149	1.187	1.156	1.141
S.E.M.	0.162	0.154	0.160	0.156


### Event-Related Potentials: ERAN and P3a Amplitudes and Latencies

**FIGURE 2:**
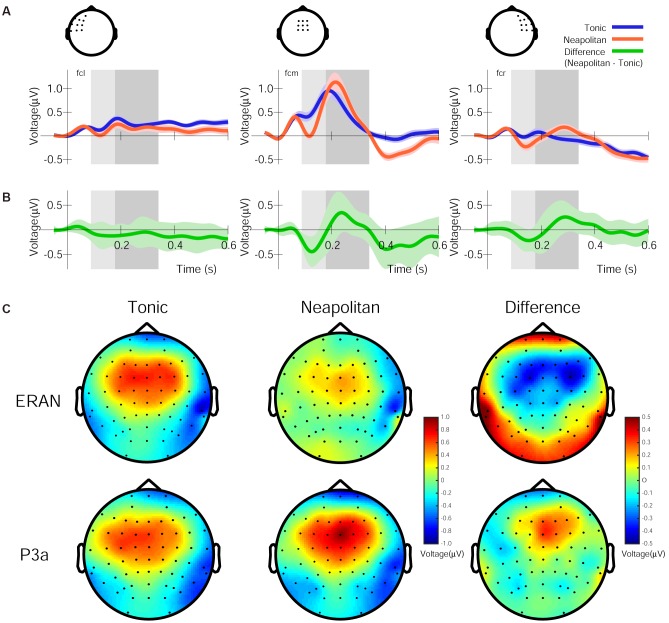
Grand average ERP responses, difference waveforms, and their topographies, averaged across all four visual conditions. **(A)** The ERP responses for the Tonic (blue line) and Neapolitan (red line) chord, in the frontocentral left, midline, and right regions, averaged over the electrodes shown in the drawing on top. Note that negative voltage is plotted downwards, and time zero refers to the onset of the fifth chord in the stimulus sequence. The lightly colored areas around the lines indicate standard error of the mean across participants. The rectangles (light and dark gray) indicate the time windows used to obtain the ERAN and P3a amplitudes, respectively. **(B)** The difference waveforms (Neapolitan – Tonic) as indicated by the green line. **(C)** Topographic maps of the amplitudes of ERPs for Tonic, Neapolitan, and Difference in the time windows for the ERAN (top row) and the P3a (bottom row) after the fifth chord onset.

Figure 2A summarizes the ERPs elicited after the onset of the fifth chord in the frontocentral left, midline and right scalp areas for Tonic and Neapolitan endings averaged across all visual cue conditions, preceding contexts, and participants. As shown in Figure 2B, the difference waveforms (Neapolitan – Tonic) commonly show a biphasic pattern from a negativity peak around 120 ms to a positivity around 230–330 ms. The topographic maps of the ERP amplitudes for Tonic, Neapolitan, and Difference in the time windows defined by half of the peak-to-peak amplitudes are shown in Figure 2C. The difference topography shows a frontocentral negativity for the ERAN time window, and a positivity for the P3a time window, both with a slight right-laterality.

The ERAN amplitudes were compared through an ANOVA with four within-subject factors of Cue-predictability (informative vs. non-informative), Cue-style (musical vs. non-musical), Previous-trial (Tonic vs. Neapolitan), and Scalp-area (Left, Midline, Right). This revealed a significant main effect of Previous-trial [*F*(1, 17) = 5.021, *p* = 0.039, $\eta_{p}^{2}$ = 0.228], caused by the increased negativity observed in Tonic-following trials compared to Neapolitan-following trials (*p* = 0.038). The main effect of Scalp-area was also significant [*F*(2, 34) = 4.815, *p* = 0.014, $\eta_{p}^{2}$ = 0.221] because the ERAN amplitude was significantly larger in the midline compared to the left (Left vs. Midline, *p_corrected_* = 0.0081). The interaction between Previous-trial, Cue-predictability and Scalp-area was significant [*F*(2, 34) = 3.548, *p* = 0.040, $\eta_{p}^{2}$ = 0.173], due to the enhanced negativity in the Tonic-following trials for the non-informative cue at the midline scalp site compared to the informative cue (*p* = 0.020), a difference that was absent in the Neapolitan-following trials.

For the P3a, the ANOVA revealed a main effect of Scalp-area [*F*(2, 34) = 5.619, *p* = 0.008, $\eta_{p}^{2}$ = 0.248], caused by larger positivities elicited in the midline area compared to the left (*p_corrected_* < 0.0001), while the midline and the right or the left and the right areas did not differ significantly. The two-way interaction between Previous-trial and Scalp-area was significant [*F*(2, 34) = 3.729, *p* = 0.034, $\eta_{p}^{2}$ = 0.180] since in the midline scalp area, the P3a was larger for Tonic-following trials than in Neapolitan-following trials while the *post hoc* test did not reach the significance level (*p* = 0.0815).

Peak latencies of the ERAN and the P3a were extracted in the midline electrode group, as indicated in Table 2. A repeated measures ANOVA with three within-subject factors, Previous-trial (Tonic vs. Neapolitan), Cue-predictability (informative vs. non-informative), and Cue-style (musical vs. non-musical), revealed no significant main effects or interactions in either the ERAN or the P3a. The simplified ANOVA results for both amplitudes and latencies are described in Table 3.

**Table 2:** Amplitude and latencies of the ERAN and the P3a in the fronto-central midline scalp area.

Previous-trial	Tonic-following				Neapolitan-following			
		
Cue-style	Non-musical		Musical		Non-musical		Musical	
				
Cue-predictability	Non-informative (audio-only)	Informative (word)	Non-informative (empty-staves)	Informative (complete-notation)	Non-informative (audio-only)	Informative (word)	Non-informative (empty-staves)	Informative (complete-notation)
ERAN amplitude (μV)	-0.497	-0.188	-0.525	-0.271	-0.296	-0.359	-0.173	-0.231
S.E.M.	0.161	0.087	0.159	0.111	0.104	0.108	0.122	0.124
ERAN latency (ms)	158.5	165.6	163.5	162.6	149.6	160.5	154.2	155.8
S.E.M.	7.4	12.1	8.2	8.8	10.6	10.9	9.3	10.9
P3a amplitude (μV)	0.128	0.516	0.115	0.323	-0.017	0.055	0.170	0.105
S.E.M.	0.159	0.172	0.189	0.138	0.149	0.149	0.126	0.126
P3a latency (ms)	356.6	357.2	370.1	304.6	323.2	381.7	295.8	310.0
S.E.M.	21.5	23.2	23.3	21.7	26.4	28.7	31.0	19.9


**Table 3:** ANOVA results for the ERAN and P3a amplitudes and latencies.

Response	Measures	Effects	*F*	*df*	*P*	$\eta_{p}^{2}$
ERAN	Amplitude	Prev	5.021	1, 17	0.039	0.228
		Scalp	4.815	2, 34	0.014	0.221
		Prev × Cue × Scalp	3.548	2, 34	0.040	0.173
	Latency	N.S.				
P3a	Amplitude	Scalp	5.619	2, 34		0.248
		Prev × Scalp	3.729	2, 34	0.034	0.180
	Latency	N.S.				


*Prev, Previous-trial; Cue, Cue-predictability; Style, Cue-style; Scalp, Scalp-area.*

### Principal Component Analysis and Comparison of the ERAN and P3a Amplitudes and Latencies

**FIGURE 3:**
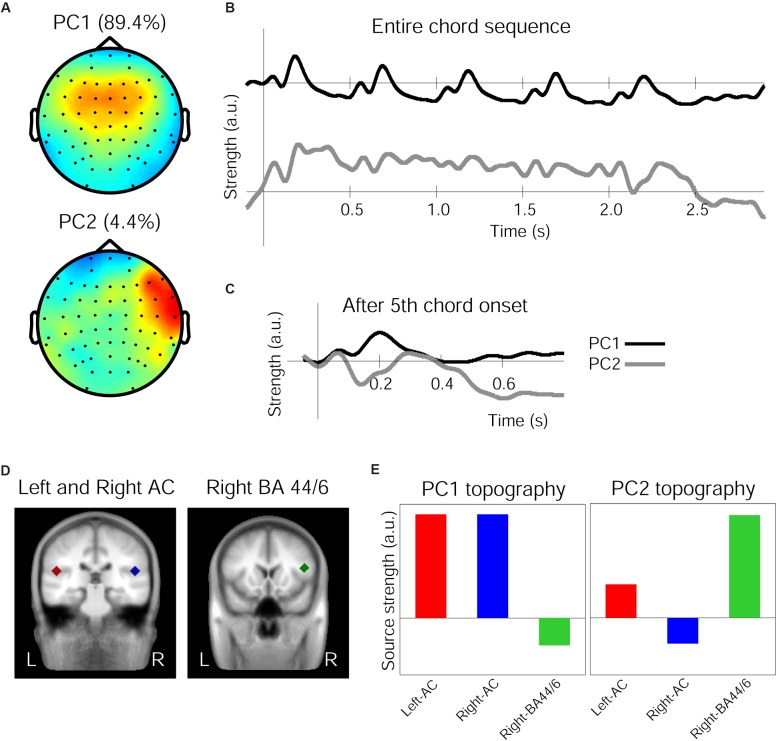
Principal components (PCs) of the grand average response in the Audio-only condition. **(A)** Topographies of the spatial filters obtained by PCA for the first PC (top) and the second PC (bottom). Note that warm colors correspond to positivity and cold colors correspond to negativity, while the scaling is based on an arbitrary unit after the decomposition. **(B)** Time courses showing the strength of the first PC (top, black line) and the second PC (bottom, gray line), depicting entire waveforms obtained by the decomposition. **(C)** Time course of the PCs after the onset of the fifth chord only, with a re-adjusted baseline and a 50 ms window beforehand. **(D)** The three regional sources used for seed-location-based source analysis, in the left-auditory cortex (AC, red carat) and right-AC (blue carat) (left panel) and the right inferior frontal cortex around BA-44/6 (green carat) (right panel), overlaid on a template-brain MRI. **(E)** Relative contribution of the three sources to the PC1 and PC2 topographies, respectively.

The 1st and 2nd PCs (henceforth PC1 and PC2) of the grand averaged response in the Audio-only condition accounted for 89.43 and 4.41% of variance, respectively. The spatial components of these PCs are shown in Figure 3A. While the PC1 topography shows a symmetrical fronto-central positivity fairly similar to the auditory evoked response, the PC2 topography shows a right-lateralized frontal positivity. The grand average response projected onto the PC1 and PC2 spatial filters are shown in Figure 3B for the entire chord sequence from the onset of the first chord (time zero). It is clear that, every 500 ms, each of the five chords elicited the characteristic triphasic P1-N1-P2-like peaks in the PC1, while the PC2 waveforms were more variable between the chord positions. In particular, the PC2 waveform exhibits a substantially enlarged negativity that peaks around 140 ms, as shown in Figure 3C, which illustrates an overlay of the two waveforms after the onset of the fifth chord.

The source analysis in BESA used three regional sources located in the left and right temporal lobes and in the right frontal area based on the aforementioned fMRI findings (Koelsch et al., 2002a), as indicated in Figure 3D and Table 4. When these three sources were applied to the Audio-only grand average evoked response (from which the PCs were derived), the GOF was 88.56%. Further, these three regional sources were applied to the PC1 and PC2 topographies to see how they contributed differently to account for each topographic map. This resulted in the dipole orientations indicated in Table 4. The GOF for PC1 was 86.59%, and for PC2, it was 81.06%. The source strength of the three sources for each topography is indicated in Figure 3E. As shown in these bar charts, the PC1 topography was mainly contributed by the bilateral temporal sources, while the PC2 topography was primarily contributed by the right frontal source. For PC1, the bilateral AC sources accounted for 85.47% of the topography, but deactivating them resulted in 19.08%. In contrast, for PC2, only the right frontal source generated a GOF of 71.29%, but deactivating it resulted in 21.06%.

**Table 4:** Source coordinates and orientations of the three sources and fitted orientations for PC1 and PC2 topographies.

	Sources	Talairach coordinate		

		X (left-to-right)	Y (posterior-anterior)	Z (inferior-superior)
	Left-AC	-48.2	-26.0	15.0
	Right-AC	49.0	-30.6	15.0
	Right-BA44/6	44.5	13.5	18.0

		**Dipole Orientation**		
		
		**Xo**	**Yo**	**Zo**

PC1	Left-AC	0.0	0.4	0.9
	Right-AC	-0.3	0.1	1.0
	Right-BA44/6	-0.3	0.6	-0.8
PC2	Left-AC	-0.8	-0.1	0.6
	Right-AC	0.2	0.1	1.0
	Right-BA44/6	0.9	-0.1	0.4


**FIGURE 4:**
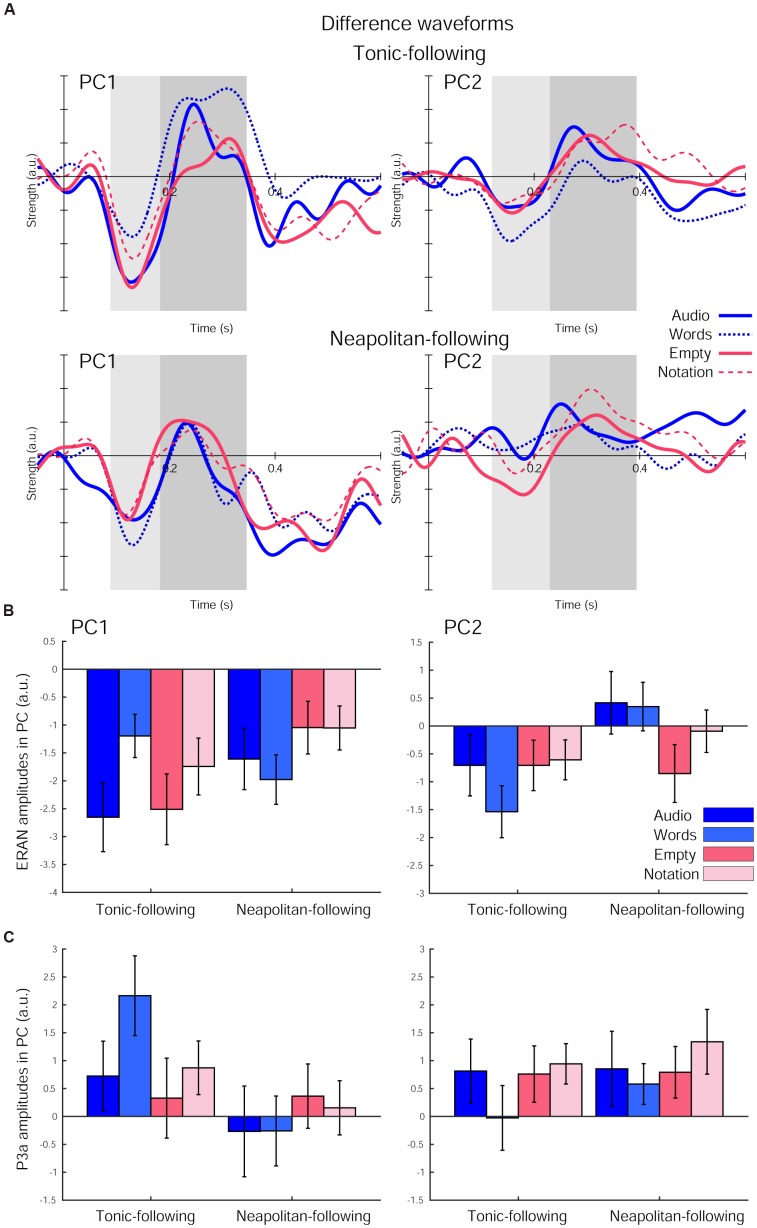
The difference waveforms (Neapolitan – Tonic) projected onto each of the two principal components (PCs) after the fifth chord onset in each of the four visual cue conditions and the two preceding context types. **(A)** The waveforms for Tonic-following trials (top) and Neapolitan-following trials (bottom) for PC1 (left) and PC2 (right). Each plot depicts the waveforms in the Audio-only (solid blue line), Word (dotted blue line), Empty Staves (solid red line), and Complete Notation (dashed red line) conditions. The shaded rectangular areas (light and dark gray) illustrate the time windows used for the ERAN and P3a amplitude comparisons, respectively. **(B)** Bar plots summarizing the ERAN amplitudes, and **(C)** P3a amplitudes for all four visual cue conditions separately for the two previous trial types, for PC1 (left) and PC2 (right). Error bars represent the S.E.M.

The ERAN responses in each of the four visual cue conditions further derived as difference waveforms (Neapolitan – Tonic) were projected onto these PCs, as shown in Figure 4A. The ERAN amplitudes in each PC computed in the time window around the grand average peak with half of the peak amplitudes are indicated in Figure 4B. These amplitude values were separately compared across visual cue conditions and preceding context types by repeated measures ANOVAs with three within-subject factors, Previous-trial (Tonic vs. Neapolitan), Cue-predictability (informative vs. non-informative), and Cue-style (musical vs. non-musical). For PC1, the ANOVA revealed no main effects, while a two-way interaction between Previous-trial and Cue-predictability was significant [*F*(1, 17) = 12.819, *p* = 0.002, $\eta_{p}^{2}$ = 0.430], reflecting a significantly greater negativity elicited by the non-informative cue conditions compared to the informative cue conditions (*p* = 0.0036) for Tonic-following trials. This difference was not observed for the Neapolitan-following trials.

For PC2, there was a significant main effect of Previous-trial [*F*(1, 17) = 6.182, *p* = 0.024, $\eta_{p}^{2}$ = 0.267], due to the larger negativity observed for the Tonic-following trials compared to the Neapolitan-following trials, where the response on average was almost absent (*p* = 0.0218). Another significant effect was a two-way interaction between Previous-trial and Cue-style [*F*(1, 17) = 4.701, *p* = 0.045, $\eta_{p}^{2}$ = 0.217]. This was because the response to musical cues was more negative compared to non-musical cues in the Neapolitan-following trials, with a marginally significant difference (*p* = 0.0827), while it was the opposite for the Tonic-following trials (N.S.). In fact, when contrasting the Previous-trial types within non-musical cues, the response was only negative for the Tonic-following trials, but not for the Neapolitan-following trials; this difference was significant (*p* = 0.0191). However, within musical cues, the response was not statistically different across Previous-trial types. No other main effects or interactions were found.

The P3a amplitudes are shown in Figure 4C. For PC1, the ANOVA yielded only a significant main effect of Previous-trial [*F*(1, 17) = 10.661, *p* = 0.005, $\eta_{p}^{2}$ = 0.385]. This was due to the larger positivity obtained for the Tonic-following trials compared to the Neapolitan-following trials (*p* = 0.0155). No other main effects or interactions were significant. For PC2, no main effects or interactions were found.

The peak latencies of the ERAN and the P3a for PC1 and PC2, indicated in the Table 5, were examined by repeated measures ANOVAs with three within-subject factors, Previous-trial (Tonic vs. Neapolitan), Cue-predictability (informative vs. non-informative), and Cue-style (musical vs. non-musical). For PC1, only the main effect of Previous-trial was significant [*F*(1, 17) = 6.605, *p* = 0.020, $\eta_{p}^{2}$ = 0.280]. This was caused by the earlier latency in the Neapolitan-following trials compared to the Tonic-following trials (*p* = 0.0491), likely related to the larger amplitude of the response for the latter. For PC2, there was a two-way interaction between Previous-trial and Cue-style [*F*(1, 17) = 6.001, *p* = 0.025, $\eta_{p}^{2}$ = 0.361], because in the Neapolitan-following trials, the ERAN peak was earlier with non-musical cues than for musical cues (*p* = 0.043) while in Tonic-following trials the latencies were comparable for non-musical and musical cues. The P3a latency results were very similar to those in the ERAN. For PC1, only the main effect of Previous-trial was significant [*F*(1, 17) = 6.329, *p* = 0.022, $\eta_{p}^{2}$ = 0.271], due to the earlier latency in the Neapolitan-following trials compared to the Tonic-following trials (*p* = 0.027), which again had a larger amplitude. For PC2, only the main effect of Cue-style was significant [*F*(1, 17) = 9.401, *p* = 0.007, $\eta_{p}^{2}$ = 0.356], caused by the later peak in the musical cue conditions than that in non-musical cue conditions (*p* = 0.002).

**Table 5:** Peak latencies of the ERAN and the P3a in the PC1 and PC2 waveforms.

Previous-trial	Tonic-following				Neapolitan- following			
		
Cue-style	Non-musical		Musical		Non-musical		Musical	
				
Cue-predictability	Non-informative (audio-only)	Informative (word)	Non-informative (empty-staves)	Informative (complete-notation)	Non-informative (audio-only)	Informative (word)	Non-informative (empty-staves)	Informative (complete-notation)
ERAN-PC1 latency (ms)	142.1	123.2	137.8	142.7	124.3	125.1	126.7	113.8
S.E.M.	8.0	8.7	8.7	7.3	9.7	6.7	11.0	6.8
ERAN-PC2 latency (ms)	166.0	159.5	157.2	160.4	147.5	139.3	154.1	165.5
S.E.M.	9.2	10.1	10.4	11.3	9.1	11.8	8.0	8.1
P3a-PC1 latency (ms)	304.0	301.6	323.2	287.3	247.0	276.4	261.6	286.8
S.E.M.	17.2	19.8	21.7	16.3	30.7	23.0	18.0	17.1
P3a-PC2 latency (ms)	297.4	328.4	352.0	351.5	320.4	290.2	356.0	343.3
S.E.M.	9.9	19.4	23.2	23.7	9.1	11.8	8.03	8.19


The simplified ANOVA results for ERAN and P3a amplitude and latency are described in Table 6.

**Table 6:** ANOVA results for the ERAN and P3a amplitudes and latencies, obtained for PC1 and PC2.

Response	Component	Measures	Effect	*F*(1, 17)	*p*	$\eta_{p}^{2}$
ERAN	PC1	Amplitude	Prev × Cue	12.819	0.002	0.430
		Latency	Prev	6.605	0.020	0.280
ERAN	PC2	Amplitude	Prev	6.182	0.024	0.267
			Prev × Style	4.701	0.045	0.217
		Latency	Prev × Style	6.001	0.025	0.361
P3a	PC1	Amplitude	Prev	10.661	0.005	0.385
		Latency	Prev	6.329	0.022	0.271
P3a	PC2	Amplitude	N.S.			
		Latency	Style	9.401	0.007	0.356


*Prev, Previous-trial; Cue, Cue-predictability; Style, Cue-style.*

## Discussion

Our musically trained participants showed slightly right-lateralized ERAN in response to Neapolitan chords as compared to Tonic chords in all visual conditions, which is consistent with previous findings and confirms the reliability of our data. Remarkably, this slightly right-lateralized but largely bilateral pattern was clearly separated into two PCs derived from the data in the Audio-only condition; one with a symmetrical frontocentral voltage concentration reminiscent of typical auditory evoked responses such as the N1 and the MMN (although their generators are overlapping but considered non-identical, e.g., Alho et al., 1986), and the other with a focal right-lateralized frontal voltage concentration. The time course of the former showed a repeated pattern of auditory evoked responses (e.g., P1-N1-P2-SP, SP: Sustained Potential) at each of the five chords with a gradual decrease in amplitude, and the latter showed a similar trend but with additional variable patterns and a disproportionately large negativity after the onset of the fifth chord. Our PCA analysis using spatio-temporal SVD (Lagerlund et al., 1997) showed that the time courses of the two components shared by electrodes with gains expressed in their respective voltage distribution topographies were mostly separable. Furthermore, our seeded-dipole source analysis confirmed that the first PC, showing a symmetrical frontocentral topography, was contributed mainly by bilateral auditory cortical sources, whereas the second PC, with its right-lateralized frontal voltage, was contributed largely by a right frontal source centered at the IFG. These two major sources have previously been found for auditory processing of chord sequences; those specific to auditory deviant processing like the MMN found in the superior temporal plane (Garza Villarreal et al., 2011), and those specific to tonal and harmonic syntax processing found in the inferior frontal cortices through MEG (Maess et al., 2001), EEG (Garza Villarreal et al., 2011), and fMRI (Tillmann et al., 2003a; Koelsch et al., 2005). It is important to note that, when listeners (musicians and non-musicians alike) are processing chord sequences that include a moving tonal center or an out-of-key chord, both of these areas are simultaneously active in their brains as shown in fMRI studies (Koelsch et al., 2002a, 2005; Tillmann et al., 2006; Bianco et al., 2016). Our data are consistent with such observation, in that the sources underlying these topographies are not necessarily perfectly isolated, but concurrently active with different dynamics throughout online chord sequence processing.

The temporal ERAN response in PC1 was the only component sensitive to the presence or absence of predictability provided by the visual cues, but this effect manifested as an interaction with the previous trial type, which was, by itself, not significant. Specifically, the ERAN in the Tonic-following trials was enhanced for non-informative cues compared to informative cues, regardless of cue style (i.e., audio vs. empty, or words vs. musical notation). In contrast, the second PC was more attenuated when preceded by Neapolitan-ending trials compared to when preceded by Tonic-ending trials, but was insensitive to cue predictability. It is important to note that the amount of ERAN reduction in PC2 was quite substantial. This supports our idea that schematic processing is online and dynamic, constantly updating schemata that are used for prediction of the next event analysis. In other words, incorporating the experience of hearing a Neapolitan-ending trial likely resulted in a diminished strength in the sense of tonality for the next trial. Our finding that this effect was specific to the frontal ERAN without the influence of cue predictability is in line with previous behavioral (Tillmann and Bigand, 2010) and neurophysiological studies (Schön and Besson, 2005; Guo and Koelsch, 2016) showing the resilience of schematic expectation processing against cue information in harmonic priming tasks. It further extends them by showing that the frontal area is responsible for forming schematic expectation from long-term knowledge as music progresses (Koelsch et al., 2000, 2004; Leino et al., 2007), as well as for updating the current status with its consequence, whether the expectation has been met or not. Interestingly, the preceding Neapolitan trial also effectively diminished the subsequent P3a in the temporal area in our data, but without the interaction with cue information. The observed asymmetries in the cue effects between the frontal and temporal components, as well as between Tonic and Neapolitan previous trial types, may be informative in understanding how schematic and veridical expectation could interact. Because our stimuli employed a 50/50 ratio between Tonic-ending and Neapolitan-ending trials, participants’ expectation for hearing a Tonic-ending sequence could have been strengthened after one or two Neapolitan-ending trials. A similar expectation should happen for a Neapolitan-ending sequence following Tonic-ending trials. However, the variability of the ERAN strength in response to cue predictability, only seen in PC1, was asymmetric between the two previous trial types. After hearing a Tonic-ending trial, a stronger sense of tonality could be formed in the frontal cortex, and conveyed to the temporal lobes where the general prediction is made by integrating the cue information. Previous findings show that the temporal area incorporates veridical expectation for producing reduced responses in ERPs such as the MMN (Todorovic and de Lange, 2012; Chennu et al., 2013). Our pattern of results suggests that the strength of the resultant tonal representation formed in the frontal area, together with the veridical information incorporated in the temporal area, may inform the early part of predictive processing as reflected in the ERAN, while the subsequent P3a-related process may operate with the expectation strength itself.

Cue style affected only the frontal ERAN, in that the reduction of the ERAN in the Neapolitan-following trials occurred only for non-musical visual cues. In contrast, when musical cues were used, the response amplitudes stayed moderate and comparable for both Tonic- and Neapolitan-following trials. The results confirm our hypothesis that musical and non-musical cues would differently affect musicians especially in the frontal activities, where tonal rules are primarily processed. Interestingly, it did not matter whether the musical cue consisted of non-informative empty staves or complete notation showing all the notes. If, as we had previously speculated, the auditory imagery formed by score reading accounts for the difference in processing of musical and non-musical cues, the ERAN reduction in the Neapolitan-following trials should have been seen in the empty-staves condition as it was in the non-musical cue conditions, since empty staves do not contain any imagery-forming note information either. Our pattern of data possibly points to a different process by which musical cues, regardless of whether they contain any actual notes, might help strengthen schematic expectations about the incoming auditory stimulus. It is possible that our musically trained participants were employing different “listening modes” when presented with musical vs. non-musical cues. Indeed, when musicians study for theory or ear-training, compose, or improvise, it is not uncommon for them to look at an empty sheet of staves. Such familiarity or attentional state in musicians may have facilitated a stronger schematic expectation, canceling out the detrimental influence of a preceding Neapolitan-ending trial. Future studies exploring this relationship may help shed light on how such generic but non-informative musical visual information could influence tonality processing in musicians. It is also important to note that our visual stimulus design introduced unbalanced amounts of visual information across the four cue types. We chose the data analysis approach based on PCA, which minimized the difference in visual-related neural activities and extracted only auditory-related responses. However, the visual processing difference itself, as well as the absence of a task related to the visual conditions, may have contributed to the pattern of results.

Regarding peak latency of ERAN, it is first important to note that, in our data, the peak occurred around 160 ms in the frontocentral midline electrode. This is largely consistent with previously reported ERAN peak latencies obtained also in the electrode space using a stimulus sequence without key changes within a block (c.f., Koelsch et al., 2000, 2001). The P3a latencies obtained in our data are also in line with the previous data, around 300–380 ms in the aforementioned studies. When sequences are transposed across different keys on a trial-to-trial basis, the ERAN peak appears to occur later around 180–210 ms (c.f., Koelsch et al., 2007; Guo and Koelsch, 2016), with slightly earlier activities (10–20 ms) in musically trained participants. When our data were projected onto the PC-based space, the latency measures between PC1 and PC2 showed separation: the peaks in PC1 occurred earlier around 110–140 ms, whereas the peaks in PC2 remained around 150–170 ms. As for the effects of the factors, while no significant results were obtained in electrode-space data, the PC-based comparison revealed that the visual cue style only mattered for the ERAN in PC2, representing right frontal source activities, interacting with the previous trial type. This interaction exactly mirrors the pattern observed in its corresponding amplitude measure. The same line further continued for the P3a latency in PC2, which showed in turn the main effect of the cue style without interaction, similarly, reflecting the more substantial response present in the musical cue conditions compared to non-musical cues. On the other hand, ERAN and P3a latency in PC1 both showed only the main effect of the previous trial type, in that the later peak was found in the Tonic-following trials compared to the Neapolitan-following trials, consistent with the more substantial response for the former. Interestingly, in either comparison, the effect of cue predictability was absent. This is a sharp contrast from the findings described in Guo and Koelsch (2016), which demonstrated a “boost” of ERAN peak latency with the predictive cues by about 6 ms in musicians and about 20 ms in non-musicians compared to the non-cue condition. We believe that this difference might have been related to the difference in the cue style as well as the transposition in auditory stimuli trials used in the two studies. Firstly, their cues were colored visual symbols which participants learned to associate them with the Tonic or Neapolitan chords in the laboratory, but such symbols must be so generic themselves that no long-term knowledge is required or interfering in learning this association. In contrast, we used real musical and linguistic cues which must have been quite familiar to our musician participants. We speculate that because they were accustomed to processing both types of cues but in clearly distinct manners, the ERAN and P3 exhibited the strong influence of visual cue styles and its interaction with the previous trial type, as expected, but this may have obscured or interfered the “facilitation” effect described in their study. Secondly, as mentioned before, their chord sequence was transposed to one of the 12 keys for each trial, unlike our stimuli staying in one key within a block. The transposition required their listeners to start over building a new tonal center from hearing the first chord of the sequence each trial. Moreover, their cue and non-cue trials were mixed within one block. Thus, the cue information may have substantially helped the brain bypass this processing overhead. Future research could use a paradigm directly comparing these different styles of cues and interaction with the transposition to capture benefits and interferences possibly interacting with musical expertise.

## Conclusion

In conclusion, the present study sought to clarify how harmonic syntactic processing supported by schematic expectations was influenced by veridical expectations. Specifically, we examined how musicians’ brains respond to violation of schematic expectation when visual cues about the identity of the last chord in a tonal sequence were made available. Further to this, we investigated how the processing in the frontal and temporal cortical areas was affected by the consequence of the schematic expectation from the previous trial type and the visual cue type. Our data, as examined through PCA and seed-based source analysis, demonstrate that the right-frontal ERAN was insensitive to veridical expectation, while it was sensitive to previous trial type, in that the ERAN amplitude was significantly smaller in the Neapolitan-following trials than in the Tonic-following trials. Such difference, however, was almost absent when musical visual cues were used. In contrast, the ERAN responses projected onto the symmetric frontocentral component associated with bilateral temporal lobe sources were significantly boosted in the absence of predictive information in the Tonic-following trials. The results support the idea that the right frontal area plays a primary role in forming schematic expectations independently from veridical expectations, as well as in updating the current status based on the outcome of the schematic expectation, which interacted with the cue style. Our data also suggest that veridical prediction processed in the temporal lobes, in conjunction with the tonal representation formed by the right-frontal area, contribute to the ultimate establishment of the listener’s auditory processing, with possible fronto–temporal interactions underlying sequential tonality processing.

## Ethics Statement

This study was carried out in accordance with the Declaration of Helsinki. All subjects gave written informed consent. The protocol was approved by the Stanford Institutional Review Board.

## Author Contributions

HS and TF designed the study, collected and analyzed the data, and wrote the manuscript.

## Conflict of Interest Statement

The authors declare that the research was conducted in the absence of any commercial or financial relationships that could be construed as a potential conflict of interest.
